# Hallmarks of the relationship between host and *Trypanosoma cruzi* sulfated glycoconjugates along the course of Chagas disease

**DOI:** 10.3389/fcimb.2023.1028496

**Published:** 2023-05-15

**Authors:** Luciana L. Soprano, Maximiliano R. Ferrero, Thomas Jacobs, Alicia S. Couto, Vilma G. Duschak

**Affiliations:** ^1^ Area of Protein Biochemistry and Parasite Glycobiology, Research Department National Institute of Parasitology (INP)”Dr. Mario Fatala Chaben”, National Administration of Health Institutes (ANLIS)-Malbrán, National Health Department, National Council of Scientific and Technical Research (CONICET), Buenos Aires, Argentina; ^2^ Max-Planck Heart and Lung Laboratory, Research Institute in Biomedicine in Buenos Aires (IBioBA), Argentine-Department of Internal Medicine II, University Medical Center Giessen and Marburg, Giessen, Germany; ^3^ Immunology Department, Bernhard Notch Institute of Tropical Medicine, Hamburg, Germany; ^4^ Faculty in Exact and Natural Sciences (FCEN), Chemical Organic Department-National Council of Scientific and Technical Research (CONICET), Center of CarboHydrates (CHIHIDECAR), University of Buenos Aires, Buenos Aires, Argentina

**Keywords:** *Trypanosoma cruzi*, Chagas disease, sulfated glycoconjugates, sulfotopes, infection, immunomodulation, immunopathogenesis, biomarkers

## Abstract

American Trypanosomiasis or Chagas disease (ChD), a major problem that is still endemic in large areas of Latin America, is caused by *Trypanosoma cruzi*. This agent holds a major antigen, cruzipain (Cz). Its C-terminal domain (C-T) is retained in the glycoprotein mature form and bears several post-translational modifications. Glycoproteins containing sulfated N-linked oligosaccharides have been mostly implicated in numerous specific procedures of molecular recognition. The presence of sulfated oligosaccharides was demonstrated in Cz, also in a minor abundant antigen with serine-carboxypeptidase (SCP) activity, as well as in parasite sulfatides. Sulfate-bearing glycoproteins in Trypanosomatids are targets of specific immune responses. *T. cruzi* chronically infected subjects mount specific humoral immune responses to sulfated Cz. Unexpectedly, in the absence of infection, mice immunized with C-T, but not with sulfate-depleted C-T, showed ultrastructural heart anomalous pathological effects. Moreover, the synthetic anionic sugar conjugate GlcNAc_6_SO_3_-BSA showed to mimic the N-glycan-linked sulfated epitope (sulfotope) humoral responses that natural Cz elicits. Furthermore, it has been reported that sulfotopes participate *via* the binding of sialic acid Ig-like-specific lectins (Siglecs) to sulfosialylated glycoproteins in the immunomodulation by host**–**parasite interaction as well as in the parasite infection process. Strikingly, recent evidence involved Cz-sulfotope-specific antibodies in the immunopathogenesis and infection processes during the experimental ChD. Remarkably, sera from chronically *T. cruzi*-infected individuals with mild disease displayed higher levels of IgG_2_ antibodies specific for sulfated glycoproteins and sulfatides than those with more severe forms of the disease, evidencing that *T. cruzi* sulfotopes are antigenic independently of the sulfated glycoconjugate type. Ongoing assays indicate that antibodies specific for sulfotopes might be considered biomarkers of human cardiac ChD progression, playing a role as predictors of stability from the early mild stages of chronic ChD.

## 1 Introduction

Chagas disease (ChD) or American Trypanosomiasis represents a major health problem and continues to be endemic in large areas of Latin America. Transmission mainly occurs by triatomine insect vectors. The causative agent is the parasitic protozoan *Trypanosoma cruzi*. It presents an intricate biological cycle in response to the accurate adaptations essential for living in an insect vector and a mammalian host. While the vector and the host present proliferative epimastigotes and intracellular amastigotes, respectively, non-proliferative infective trypomastigote forms are involved in the two mentioned hosts. At present, the number of infected people globally estimated by the World Health Organization (WHO) sums 6 to 7 million, and approximately 70 million people live at risk of contracting the disease (WHO, 2021). This neglected disease is endemic in 21 countries, although the migration of infected people can transport it to non-endemic countries of America (PAHO, 2021). In the last decades, the mentioned increase in the migratory flow as well as urbanization has spread the disease to other continents (WHO, 2021). Also, parasite transmission by people migrating to other regions of the world such as the USA, Europe, Australia, and Japan has determined its emergence as a public health problem in all non-endemic countries (Gascon et al., 2010; Pérez-Molina et al., 2012). The problem is amplified by the fact that most of the existing therapeutics were discovered decades ago and suffer from acute toxicity and little extent of action. The increase in the understanding of the biology and biochemistry of the parasite has allowed the identification of new therapeutic targets to identify new trypanocidal agents in addition to rational drug design and screening of natural products. Existing drugs are active in treating congenital infection by *T. cruzi*, acute ChD, and children up to 12 years. Little evidence exists related to the effectiveness of treatment in adults. Several clinical trials have shown that benznidazole is effective as a treatment. The problem is the timing and how to evaluate seroconversion. In addition, new active trypanocidal compounds, with low toxicities and increased efficacies during the chronic phase, are urgently needed (Duschak, 2011; Duschak, 2016; Duschak, 2017; Duschak, 2019). ChD is the most important form of infectious myocarditis (Tarleton, 2015). Approximately 30%–50% of infected individuals will develop from mild to severe cardiac alterations, and it is a shared cause of fatal dilated cardiomyopathy (Marin-Neto et al., 2007; Andrade et al., 2011; Sabino et al., 2013), while the compromise of the intestinal wall is less frequent. The pathogenesis of ChD is debatable, but irrespective of the eventual influence of an autoimmune component (Leon and Engman, 2001; Leon and Engman, 2003; Cunha-Neto et al., 2006), pathology has been related to the persistence of *T. cruzi* parasites in the affected organs where they induce chronic inflammation (Tarleton, 2001). Likewise, it has been proposed that the persistence of the parasite in conjunction with immune responses against multiple myocardial antigens might contribute to heart damage (Gironés and Fresno, 2003; Gironés et al., 2007) but whatever the cause of the pathogenesis, cruzipain (Cz), the major cysteine proteinase (CP) of this flagellate parasitic protozoan, has been considered as a relevant factor for the disease progression.

### 1.2 *Trypanosoma cruzi* post-translational modifications

#### 1.2.1 Proteolytic activities: proteinases/peptidases

The multiple roles attributed to *T. cruzi* proteinases have turned these proteins into relevant potential targets for the development of novel agents to fight against ChD (Cazzulo, 2002; Duschak, 2011; Duschak, 2016; Duschak, 2017; Duschak, 2019). After the complete sequence of *T. cruzi* clone CL Brener genome, cysteine peptidases (CPs), serine peptidases (SPs), metallopeptidases (MPs), threonine peptidases (TPs), and only two aspartic peptidases (APs) were predicted (El-Sayed et al., 2005). A summary of the CPs detected in *T. cruzi*, in accordance with the Genome Project and the MEROPS database, has been described in detail (Rawlings and Bateman, 2021). Briefly, several types of CPs corresponding to different clans, most of them integrated into either clan CA or CD, have been mentioned. Among those pointed out, the experimentally characterized have been mostly described: Cruzipains 1 and 2 and the cathepsin B-like peptidases corresponding to family C, the metacaspases corresponding to family C14, and the autophagins corresponding to family C54 (Alvarez et al., 2012). Among CPs, the most relevant group studied by most authors is that corresponding to Cz. This molecule has been involved with parasite metabolic rate (Klemba and Goldberg, 2002), identified as both an important candidate for vaccine development (Schnapp et al., 2002; Cazorla et al., 2009; Cerny et al., 2016), or taking part in a new chimeric antigen designed to exhibit B- and T-cell epitopes within strategic parasite protein targets (Sanchez Alberti et al., 2017; Bivona et al., 2020) as well as promising targets for therapeutics of ChD (Cazzulo, 2002; Duschak, 2011; Duschak, 2016; Duschak, 2017; Duschak, 2019) and extensively studied as protease and antigen. It has also been examined as glycoprotein (Duschak and Couto, 2009).

On the other hand, (i) oligopeptidase B, a member of the prolyl oligopeptidase family, is involved in Ca^2+^-signaling during mammalian cell invasion (Burleigh and Andrews, 1995; Burleigh et al., 1997); (ii) a prolyl endopeptidase (*Tc*80) (Santana et al., 1997; Bastos et al., 2005); and (iii) a lysosomal serine carboxypeptidase (SCP) (Parussini et al., 2003), all purified and partially characterized from *T. cruzi*, can be pointed out among the SPs described in the parasite. Also, a supposed SP, a secreted 75-kDa *T*. *cruzi* serine oligopeptidase was purified and the subcellular localization was delimited to intracellular structures, including the flagellar pocket, plasma membrane, and cytoplasmic vesicles resembling reservosomes (da Silva-Lopez et al., 2008).

It is worth noting that some peptidolytic activities such as the major parasite leucyl aminopeptidolytic activity (LAP*Tc*), a 330-kDa homohexameric metalloaminopeptidase, and an SCP were detected in cell-free extracts and lysosomes of *T. cruzi*, respectively (Parussini et al., 2003; Cadavid-Restrepo et al., 2011). Peptidases that cleave the C-terminal amino acid residue from peptides and proteins are known as carboxy-peptidases. They are categorized into two major classes, MPs and SPs, centered on their catalytic mechanism. Interestingly, peptidases displaying C-terminal proteolytic activity, in acidic conditions, are named SCPs. Esterase and deamidase activities are also present (Breddam, 1986; Remington and Breddam, 1994). In general, SPs fit into the Clan SC of the S10 family (Barrett et al., 2004). All the SCPs contain sequences neighboring the highly conserved Ser and His residues from the active site and show various common features. Among them, they share the signal sequence and have several N-linked glycosylation sites, as well as four conserved regions that participate in the catalytic mechanism (Vendrell and Avilés, 1999). The characterization of primers capable of specifically detecting *T. cruzi* DNA and the presence of intergenic regions usually divergent in Trypanosomatids in conjunction with the SCP gene were effective for searching promising species-specific markers for the identification of intra-specific polymorphisms to be used as a target for diagnostic methods by PCR and for the progress of novel approaches to detect *T. cruzi* infection (Magalhães Ferreira, et al. 2014).

#### 1.2.2 Glycosylation

Among CPs, natural Cz (Cazzulo et al., 2001), commonly showing a complex expression of a mix of isoforms, presents micro-heterogeneities as detected by chromatographic and electrophoretic methods (Cazzulo et al., 1995; Duschak et al., 1999). These micro-heterogeneities are most likely attributed both to the coinciding expression of numerous genes codifying for Cz, which contain some amino acid replacements (Campetella et al., 1992), and to the variability of post-translational modifications (PTMs) present in Cz molecules. The mentioned glycoprotein contains approximately 10% of carbohydrates and their oligosaccharidic chains do not contain phosphate groups (Cazzulo et al., 1990). The molecule bears three possible N-glycosylation sites, two of them are located in the catalytic domain, and the first one of these sites contains oligosaccharides of the high mannose (HM) type. The third N-glycosidic site is located in the Asn_255_ of the C-T domain of Cz and it is composed of HM, hybrid monoantennary or complex biantennary oligosaccharidic chains (Parodi et al., 1995; Barboza et al., 2005).

Along with the Cz lysosomal isoforms, we have reported evidence of some membrane-bound CP isoforms, immunologically cross-reactive with Cz in the different parasite developmental forms (Souto-Padron et al., 1990; Parussini et al., 1998). Also, it has been shown that metacyclic trypomastigotes are able to release CPs to the extracellular medium, involving most probably Cz among them (Duschak et al., 2006). In addition to these differences in localization, peculiar isoforms with different carbohydrate content, which do not adsorb to Concanavalin-A affinity columns (NACrI) (Duschak et al., 2003), were found. Together with the catalytic moiety, this enzyme bears a carboxy-terminal extension (C-T) that is preserved in the mature protein. This infrequent extension contains several PTMs in both natural and experimental infection; the immune-dominant antigenicity of the molecule has been attributed to this domain (Scharfstein et al., 1983; Martinez et al., 1991; Martinez et al., 1993). Our vast experience working with proteinases in *T. cruzi*, especially Cz, together with the use of more sensitive techniques, allowed us to describe in detail the presence of main PTMs, previously not found in this molecule. These PTMs include complex N-glycosydic-oligosaccharide-sialic-acid-containing structures and unique units of N-acetyl-D-glucosamine residues with an O-glycosydic linkage (O-GlcNAc) (Barboza et al., 2003). Taking into account that the sialylation process is a *T. cruzi* surface reaction and that membrane-bound isoforms of Cz were found, the identification of the presence of sialic acid in the C-T domain of Cz was very interesting. *T. cruzi* is unable to synthesize sialic acids *de novo* and has evolved an innovative metabolic pathway by which protective sialic acid residues are scavenged from host sialyl glycoconjugates from target cells and transferred onto parasite surface glycoconjugates by means of an exclusive trans-sialidase (TS) enzyme that is anchored to the surface of the parasite by a glycosylinositolphospholipid (GPI). The parasite enzyme (*Tc*TS) functions through a covalent sialyl-enzyme intermediate, being a specific tyrosine residue (Tyr_342_) the cataytic nucleophile. Acquisition of the sialyl residue allows the parasite to survive in blood and spread the infection (Watts et al., 2003; Mucci et al., 2017).

O-GlcNAcylation is a dynamic PTM of proteins located in the nucleus, cytoplasm, and mitochondria, relevant for diverse cellular processes, which is believed to control protein functions in an equivalent way to protein phosphorylation. The interaction between both PTMs allows the regulation of cellular functions in response to stress and nutrient levels (Bond and Hanover, 2015). Protein O-GlcNAcylation has been described in multicellular organisms and in some prokaryotic cells, but their occurrence in protists is a vacant field. Regarding *T. cruzi* epimastigotes, although we have previously reported N-acetyl-D-glucosamine residues with an O-glycosydic linkage (O-GlcNAc) in the C-T of Cz (Barboza et al., 2003), some years ago, another research group have identified approximately 1,200 putative O-GlcNAcylated proteins and six modification sites by mass spectrometry, confirming the occurrence of the O-GlcNAc modification in *T. cruzi* (Torres-Gutiérrez et al., 2019). Unfortunately, this is not the first time as they claim, because we had described it for the first time in 2003.

### 1.3 Sulfation

**Figure 1:**
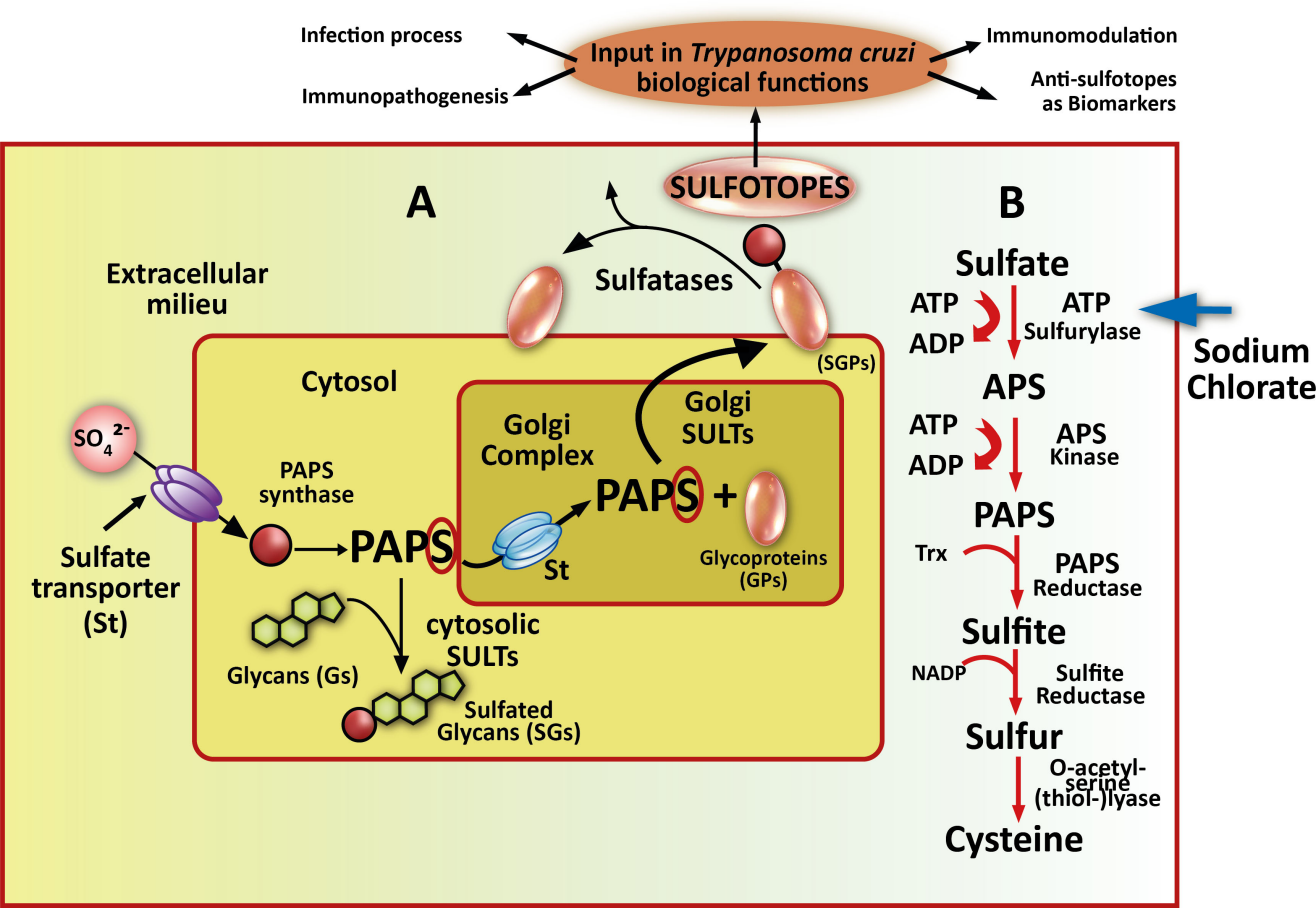
Sulfotopes in glycoproteins and PAPS route. **(A)** In sulfation pathways, parasites probably take up inorganic sulfate from the extracellular milieu through sulfate channels commonly named sulfate transporter (St). PAPS synthase is the enzyme that catalyzes the formation of 3′-phosphoadenosine 5′-phosphosulfate (PAPS) from ATP and $SO_{4}^{2-}$ in the cytosol. PAPS represents the worldwide sulfate donor for wholly described sulfotransferases (SULTs). This enzyme may be used by cytosolic SULTS when sulfation of small metabolites, such as steroids (Sds), is required but can also be translocated into the Golgi compartment through a sulfate transporter (St) when sulfation of glycoproteins (GPs) is needed. **(B)** In this sense, Golgi-localized SULTs then transfer sulfate from PAPS to emerging glycoproteins (GPs) or glycolipid substrates as they cross through the Golgi complex (this step may be followed by a presentation on the plasma membrane or secretion). The resultant sulfotopes often create specific binding epitopes for soluble factors involved in cell-to-cell communication or adhesion molecules. In certain circumstances, membrane-associated or extracellular sulfatases probably control the biological function of these parasite sulfotopes by the deletion of a minor group of critical sulfate residues. In the right part of the scheme, the PAPS metabolic route is shown, PAPS is the donor of sulfate groups necessary for the development of the sulfation process through SULTs. It is signed the KEY enzyme where sodium chlorate acts as an inhibitor with an arrow. In the upper part, the sulfated moieties of the glycoproteins are shown as sulfotopes. The arrows indicate several biological functions where sulfotopes or their specific antibodies are involved.

Sulfation has been frequently identified as an essential and ubiquitous biochemical reaction that modifies a wide range of endogenous molecules in a diversity of organisms, from prokaryotes to multicellular species. This PTM plays key roles in several biological processes including cell communication, growth, and development (Kovensky, 2009; Leung et al., 2016; Langford et al., 2017; Günal et al., 2019). The sulfation pathways are catalyzed by two types of enzymes, the sulfotransferases (SULTS) and the sulfatases (STs). Cytosolic SULTs catalyze sulfation and are involved in detoxification, hormone regulation, and drug metabolism. The membrane-associated SULTs are implicated in the sulfation of complex carbohydrates and proteins, and have appeared as essential enzymes in a number of molecular-recognition events and biochemical signaling pathways (Kakuta et al., 1998; Hemmerich and Rosen, 2000; Honke and Taniguchi, 2002; Chapman et al., 2004; Kawashima, 2006). The SULTs transfer a sulfuryl group from the universal donor PAPS (3′-phosphoadenosine 5′-phosphosulfate) to the hydroxyl or amino group of several compounds (Strott, 2002; Coughtrie, 2016). Multiple endogenous compounds including carbohydrates, lipids, proteins, and hormone precursors (steroids) are also modified by SULTs. Thus, sulfation has a significant influence on the biological activity of the modified molecules and consequently in multiple biological processes (Strott, 2002; Müller et al., 2015; Leung et al., 2016). Also, sulfated N-linked oligosaccharide-containing glycoproteins have been mostly associated with definite molecular recognition procedures (Kawasaki et al., 2000; Honke and Taniguchi, 2002). In the Golgi complex, the sulfation reaction occurs through the transference of an active sulfate group from the cytosolic PAPS (Klaassen and Boles, 1997) towards an exact location on a variety of carbohydrate residues existing in numerous substrate acceptors (Esko and Lindahl, 2001; Fukuda et al., 2001). Herein, we refer in particular to glycoproteins (**Figures 1A, B**). Sodium chlorate treatment inhibits a crucial enzyme in PAPS synthesis, the ATP sulfurylase (Baeuerle and Huttner, 1986) (**Figure 1**, right part). The sulfatases, on the other hand, hydrolyze the sulfate ester bonds to the unconjugated form of the substrate (Hanson et al., 2004) (**Figure 1**).

Regarding *T. cruzi*, we have demonstrated the presence of sulfated groups as components of epimastigote glycoproteins indicating that the parasite contains SULT activity. However, in Trypanosomatids, no data about the whole sulfation mechanism and/or SULTs have been reported so far. It is worth mentioning that there has been no matching sequence when comparing the *T. cruzi* genome databank with the described SULTs to date, although there are ongoing assays in our research group. Our findings suggested that the *T. cruzi* sulfation process takes place employing PAPS, which proved the presence of sulfated epitopes (sulfotopes) in the parasite clinical stages, confirming their existence on the surface of trypomastigotes (Ferrero et al., 2014). The comparison of amino acid sequences of different membrane and cytosolic SULTs has shown two consensus sequence zones (5´PSB and 3´PB). A high degree of conservation of substrate-binding domains among the SULTs cloned (Honke et al., 1997) has been shown earlier. Regarding the *T. cruzi* genome, there are no sequences with homology with SULTs. However, there is homology with a reductase enzyme of the PAPS synthesis route, confirming that, in *T. cruzi*, the sulfation process occurs *via* PAPS.

#### 1.3.1 Characterization of N-linked oligosaccharides in the *T. cruzi* glycoproteins cruzipain and serine carboxypeptidase: Identification of sulfated oligosaccharides

**Figure 2:**
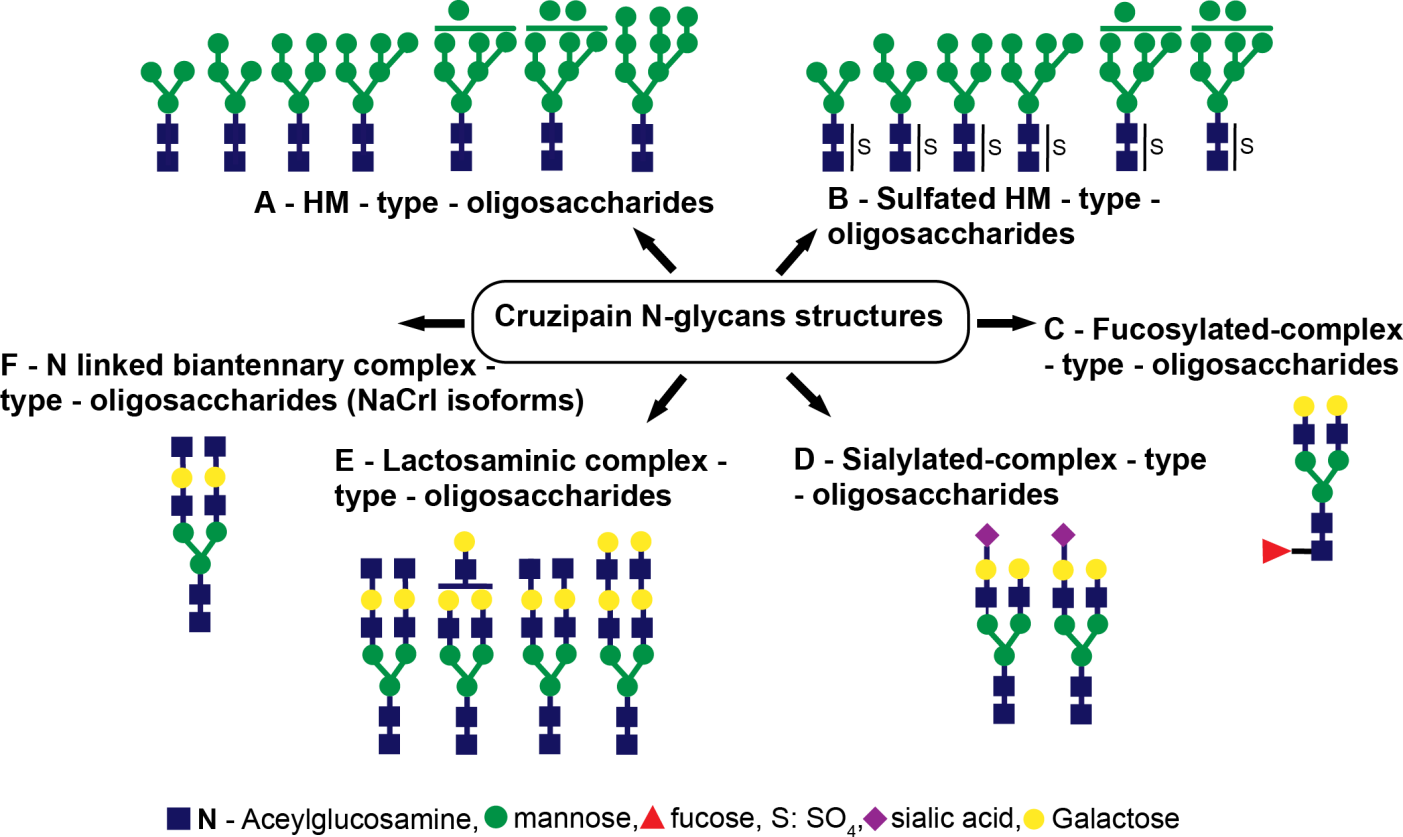
Cruzipain (Cz) N-glycan structures. The structures identified were **(A)** HM-type oligosaccharides, **(B)** sulfated HM-type oligosaccharides, **(C)** fucosylated complex-type oligosaccharides, **(D)** sialylated complex-type oligosaccharides, **(E)** lactosaminic complex-type oligosaccharides, and **(F)** N-linked biantennary complex-type oligosaccharides (NACrI isoforms).

Using UV-matrix assisted laser desorption/ionization-time of flight-mass spectrometry (UV-MALDI-TOF-MS), in conjunction with enzymatic digestions followed by high pH anion exchange chromatography (HPAEC) examination, a complete structural analysis of the oligosaccharide-linked to Asn from Cz revealed GlcNAc_2_Man_3_ to GlcNAc_2_Man_9_ species (**Figure 2A**) and, for the first time, the occurrence of sulfated HM-type-oligosaccharides (**Figure 2B**) on the single N-glycosylation site (Asn**_255_**), located in the C-T of Cz (Barboza et al., 2005). Moreover, as an interesting feature, we also detected fucosylated oligosaccharides (**Figure 2C**), structures corresponding to monosialylated species (**Figure 2D**), and lactosaminic complex glycans (**Figure 2E**) by UV-MALDI-TOF-MS analysis. The occurrence of the deoxy-sugar was established by HPAEC (Barboza et al., 2005).

**Figure 3:**
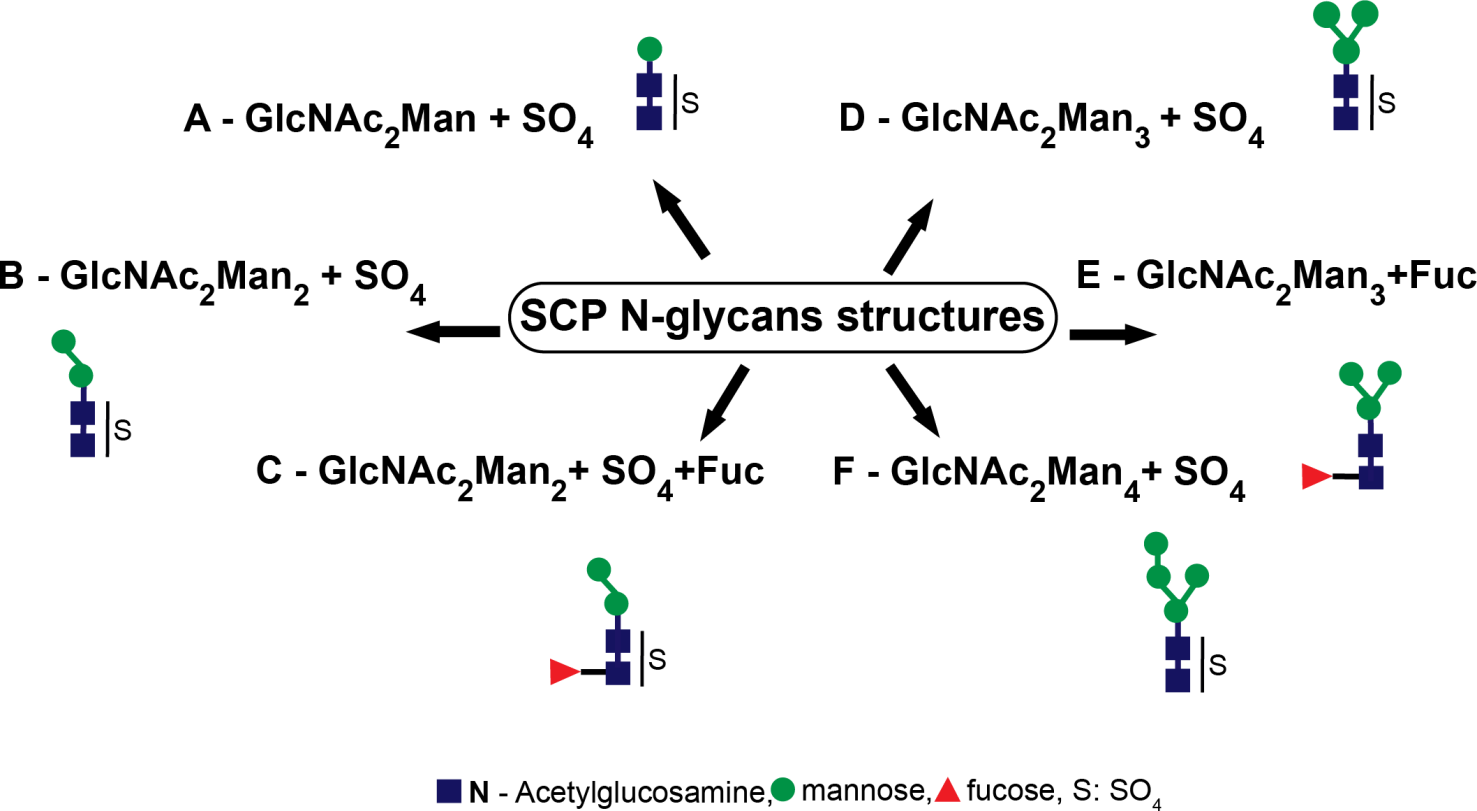
Serinecarboxipeptidase (SCP) N-glycan structures. The structures identified were **(A)** GlcNAc_2_Man+SO_4_, **(B)** GlcNAc_2_Man_2_+SO_4_, **(C)** GlcNAc_2_Man_2_Fuc+SO_4_, **(D)** GlcNAc_2_Man_3_+SO_4_, **(E)** GlcNAc_2_Man_3_Fuc, and **(F)** GlcNAc_2_Man_4_+SO_4._.

Furthermore, we also performed a mass spectrometry examination of the SCP of *T. cruzi* epimastigotes. The occurrence of sulfate groups in short N-glycosidic chains as well as fucosylated and non-sulfated oligosaccharidic structures was demonstrated (**Figure 3**).

### 1.4 Immunopathogenic properties of sulfated moieties in *Trypanosoma cruzi* glycoproteins

**Figure 4:**
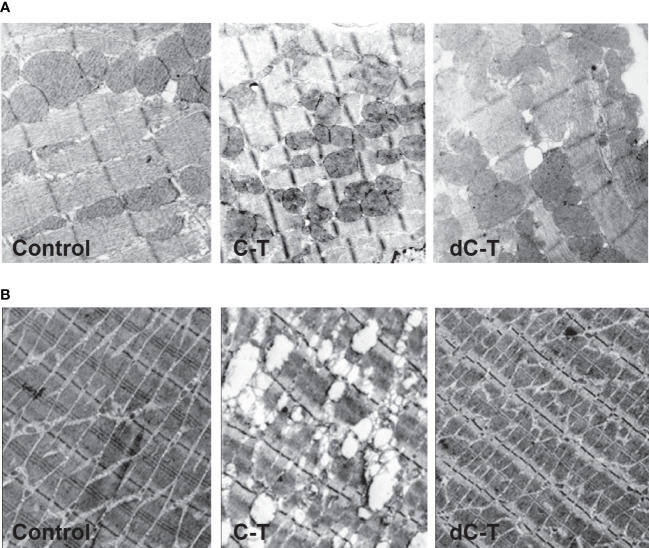
Ultrastructural features found in cardiac **(A)** and skeletal **(B)** muscle from C-T- and dC-T-immunized BALB/c mice. Morphologic analysis on mice hearts and skeletal muscle tissue was carried out by transmission electron microscopy. The control mice (left panels) presented a normal pattern in longitudinal heart sections (36,000×); severe anomalies were observed in mice immunized with C-T (central panels) and dC-T-immunized mice (right panels) exposed to a normal pattern like controls. Representative results were obtained using groups formed by three mice, working in three independent experiments. Disorganization of the regular structure is observed in C-T immunized tissues.

Our findings have demonstrated that sulfated motifs are completely necessary for producing IgG_2b_ isotype humoral immune mice responses to Cz. Moreover, in Trypanosomatids, sulfate-containing glycoproteins are targets for specific immune responses (Acosta et al., 2008). To our knowledge, it constituted the first report showing that the antigenic extension (C-T) of the Cz, when used as immunogen, is able to generate ultrastructural abnormalities in cardiac muscle tissue in the absence of infection. Surprisingly, the abolition of the observed pathological alterations that occurred in mice immunized with sulfate-depleted C-T, confirmed by electron microscopy, supports the participation of sulfotopes in the generation of experimental tissue injury (**Figure 4**). On the other hand, immune-gold-electron microscopy analysis of cardiac tissue samples from C-T-immunized mice tested with polyclonal rabbit sera specific for Cz and C-T before and after myosin adsorption established that Cz and either myosin or other cardiac O-GlcNAc-containing-proteins share O-GlcNAc moieties as common epitopes, starting a novel vision into the molecular immune pathogenesis of cardiac ChD (Acosta et al., 2011).

Cz has been defined as an HM-type glycoprotein. Furthermore, the high-mannose receptor (HMR) possesses two parts with dissimilar carbohydrate recognition, with the ability to bind to sulfated-containing and non-sulfated oligosaccharidic chains (Liu et al., 2001). Considering that the HMR is expressed by antigen-presenting cells (APCs) in non-lymphoid peripheral organs, comprising muscle of murine heart (Linehan et al., 2005), we speculate that sulfotopes present in the C-T extension might influence the HMR recognition in target cells contributing to tissue damage. Moreover, it has been postulated that Cz might favor arginase induction and intracellular parasite survival in macrophages, through the enhancement of its contact with HMR increasing its recycling (Garrido et al., 2011).

The antigenicity and immunogenicity of Cz have been extensively described. Murine and human infections with *T. cruzi* trigger an intense immune response to Cz (Murta et al., 1988; Martinez et al., 1991; Giordanengo et al., 2000; Giordanengo et al., 2002; Schnapp et al., 2002). Furthermore, we have previously reported an association between the humoral immune response to Cz and the severity of chronic ChD (Duschak et al., 2001). In addition, Cz-induced antibodies could bind to mouse heart extracts, suggesting a pathogenic role for this molecule (Giordanengo et al., 2000; Giordanengo et al., 2002). However, divergent information linked to the immunopathological belongings of Cz has been shown (Giordanengo et al., 2002; Schnapp et al., 2002). Interestingly, Gea and co-workers evaluated the impact of the genetic background on the activation of B cells by using Cz as an antigen, and showed discrepancies in modulation between C57BL/6 and BALB/c mouse strains. Significant differences in the resistance or in the pathogenic degree of the experimental disease were also described (Guiñazú et al., 2004; Pellegrini et al., 2011). Unfortunately, in the mentioned assays, the presence of a contaminant SCP that co-purifies with Cz was involuntarily present in the purification protocol of Cz (Labriola et al., 1993) used as an antigen, as we have evidenced (Acosta et al., 2008). It has been described that Cz is an antigen that bears a vastly immunogenic area (C-T), whose function is still unidentified, which perhaps must care for the catalytic domain and is vital for parasite survival. Nevertheless, the capacity of Cz to avoid responses directed towards the catalytic domain after immunization by using the N-terminal domain as an immunogen capable of refocusing host immune responses and providing improved protection was described (Cazorla et al., 2010).

As explained, several studies on Cz immunogenicity were performed using recombinant Cz as an immunogen, where sulfated epitopes are absent, so they do not reflect the effects of sulfation over the molecule immunological properties (Cazorla et al., 2009). Therefore, it is not known whether these effects occur with the natural sulfated oligosaccharides containing molecule. On the other hand, an analysis of the common epitopes between the sulfated *T. cruzi* glycoproteins, Cz and SCP, studied to date considered the antigenicity of sulfated groups (Soprano et al., 2018a). We took advantage of the availability of the two known sulfated glycoproteins, Cz and SCP, and the polyclonal rabbit anti-Cz and anti-SCP sera, to study the immune cross-reactivity between both molecules, finding the presence of common epitopes between them. Moreover, the cross-reactivity observed was attributed to the sulfate groups located in both molecules by using the desulfated Cz-SCP mix. Interestingly, *T. cruzi* SCP, an HM-type glycoprotein, which co-elutes with Cz from Concanavalin-A lectin affinity columns, was further purified and short chains containing sulfated oligosaccharides were identified by UV-MALDI-TOF-MS endorsing the involvement of sulfotopes in the cross-reactivity between both molecules (Parussini et al., 2003; Acosta et al., 2008; Soprano et al., 2018a). Regarding natural infection, when SCP was tested with the infected patient’s sera with a dissimilar degree of heart dysfunction, despite the fact that most sera recognized the glycoprotein in the groups evaluated, it was not possible to determine any statistical relationship between the severity of the disease and the presence of anti-SCP antibodies in patient sera. Remarkably, we have demonstrated that SCP is a minor abundant immunogen recognized in most chronic ChD patients (Soprano et al., 2018a). Sulfated oligosaccharides have been reported to play a variety of roles necessary for development, homeostasis, and differentiation, among others. The involvement of sulfated carbohydrates in the interaction with sugars, for glycosaminoglycan to glycosydic protein chains, and their possible use as novel targets for therapeutic agent/diagnosis and/or vaccine applications have been studied and reviewed in detail (Fukuda et al., 2001; Kovensky, 2009). In some cases, the binding of growth factors to receptors (Rapraeger et al., 1991) or adherence of the virus to the cellular surface (Shukla et al., 1999) was the role revealed. Sulfated structures of *T. cruzi* have been defined as components of glycolipids sharing antigens between the parasite surface and cells from mammals (Petry et al., 1988; Vermelho et al., 1997). In viruses (Bernstein and Compans, 1992) and particularly in mammals (Noguchi and Nakano, 1992; Van Rooijen et al., 1998; Kawasaki et al., 2000), sulfate groups in N-linked oligosaccharides have been described. Sulfated Asn-linked oligosaccharides have been predominantly associated with numerous definite recognition mechanisms (Noguchi and Nakano, 1992; Honke and Taniguchi, 2002). Noticeably, in *T. cruzi*, we have shown the antigenic properties of these structures in glycoconjugates and cross-reactivity between sulfatides and Cz was determined (Kovensky, 2009; Acosta et al., 2012). Until the discovery of the trypanosomatid sulfotope antigenic properties, the only sulfated HM-type glycans with similar properties described have been found in components of the lysosomal hydrolases from *Dictyostelium discoideum* (Freeze et al., 1984).

### 1.5 Chemical and immunological characterization of Cz sulfotope

**Figure 5:**
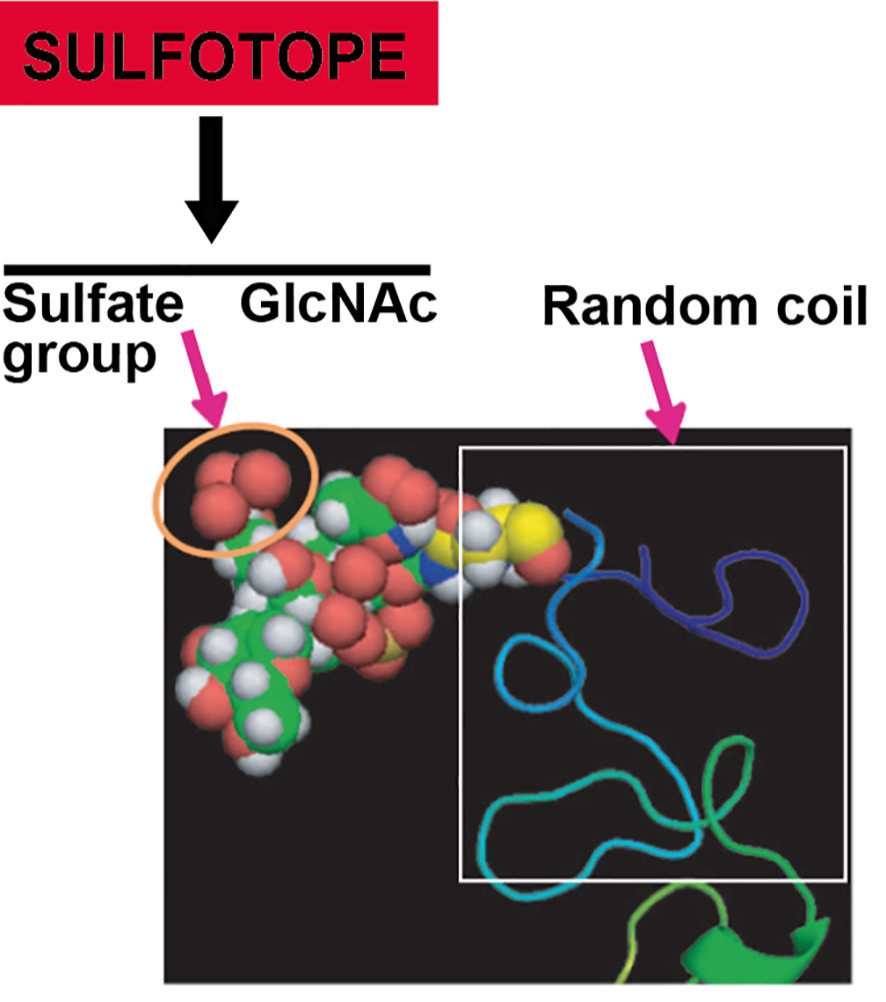
Modeling showing the exposition of the sulfotope localized in the C-T of Cz. Sulfate group (marked with an orange circle) of the oligasaccharidic chain in linkage with the Asn_255_ located in the C-T of natural Cz according to data: AAB41119.1 from GenBank. Predicted C-T domain x-ray crystal structures were obtained by an automated relative modeling program PHIRE sequence server. The Protein Data Bank presenting the nearby template allowed generating a sugar-containing model. Asn_255_ localized in a secondary structure (random coil) helps to expose the sulfated oligosaccharide (SULFOTOPE) as an epitope displaying antigenicity. The model used was based on homology and was carried out by means of MACPYMOL (PDB clautl). The figure shows an oligosaccharide GlcNAc-GlcNAc-Man containing a sulfate group (C: yellow; H: white; N: blue; O: red; SO_3_: brown) with linkage to the Asn (model as a sphere), enclosed in a secondary structure portion of the C-T, in a random coil form.

Resulting studies exhibited that Gal-6-SO_4_ and NAcGlc_6_-SO_4_ are the two specific modifications existing on genuine biological ligands (Hemmerich and Rosen, 2000; Kawashima, 2006). Regarding the chemical requirements for antibody–Cz binding, a methodical structural and immunological study was carried out in our research group with the aim of outlining the structural supplies required for the antibody recognition of Cz. In this sense, diverse molecules obtained by chemical synthesis were coupled to different carrier proteins (BSA and aprotinin). Then, they were incubated with (i) mice sera specific for Cz and C-T, (ii) anti-Cz and anti-C-T polyclonal rabbit sera, and (iii) IgGs purified from sera of patients with ChD. Interestingly, we evidenced that the preferred epitope for immune recognition was constituted by an N-acetyl glucosamine unit bearing a sulfate group in position O-6. By extending our findings to the context of natural infection, using ChD patients’ serum, we confirmed that synthetic GlcNAc_6_SO_3_ mimics the N-glycan-linked sulfotope exhibited in the C-T extension of Cz, which supports the input of this sulfated sugar in the human humoral response (Couto et al., 2012). **Figure 5** shows a model of the C-T domain of native Cz, obtained by using the Server Phire, comparing the sequence homology with other proteins. The exposition of the sulfotope located in the C-terminal extension of the Cz molecule can be observed. The sulfate group (noted by an orange circle) in the oligosaccharidic chain is linked to Asn_255_ present in the C-T of natural Cz (GenBank Data: AAB41119.1). The predicted C-T domain x-ray crystal structures were obtained by an automated relative modeling program PHIRE sequence server. The Protein Data Bank presenting the nearby template allowed generating a sugar-containing model. Asn_255_ localized in a secondary structure (random coil) helps to expose the sulfotope (sulfated oligosaccharide) as an antigenic epitope. The analysis of the homology-based model was performed using MACPYMOL (PDB c1autl). In the C-T, the Asn_255_ (Sphere model) corresponding to the consensus sequence is confined to a random coil that favors the sulfate oligosaccharide (sulfate group + GlcNAc) exposition as an antigenic epitope or SULFOTOPE.

Sulfotopes are involved in different functions such as the infection process, immunopathogenesis, and immunomodulation, and their specific antibodies are being studied in our research group as potential biomarkers of cardiac ChD progression.

### 1.6 Sulfotopes involvement in the infection by *Trypanosoma cruzi*

**Figure 6:**
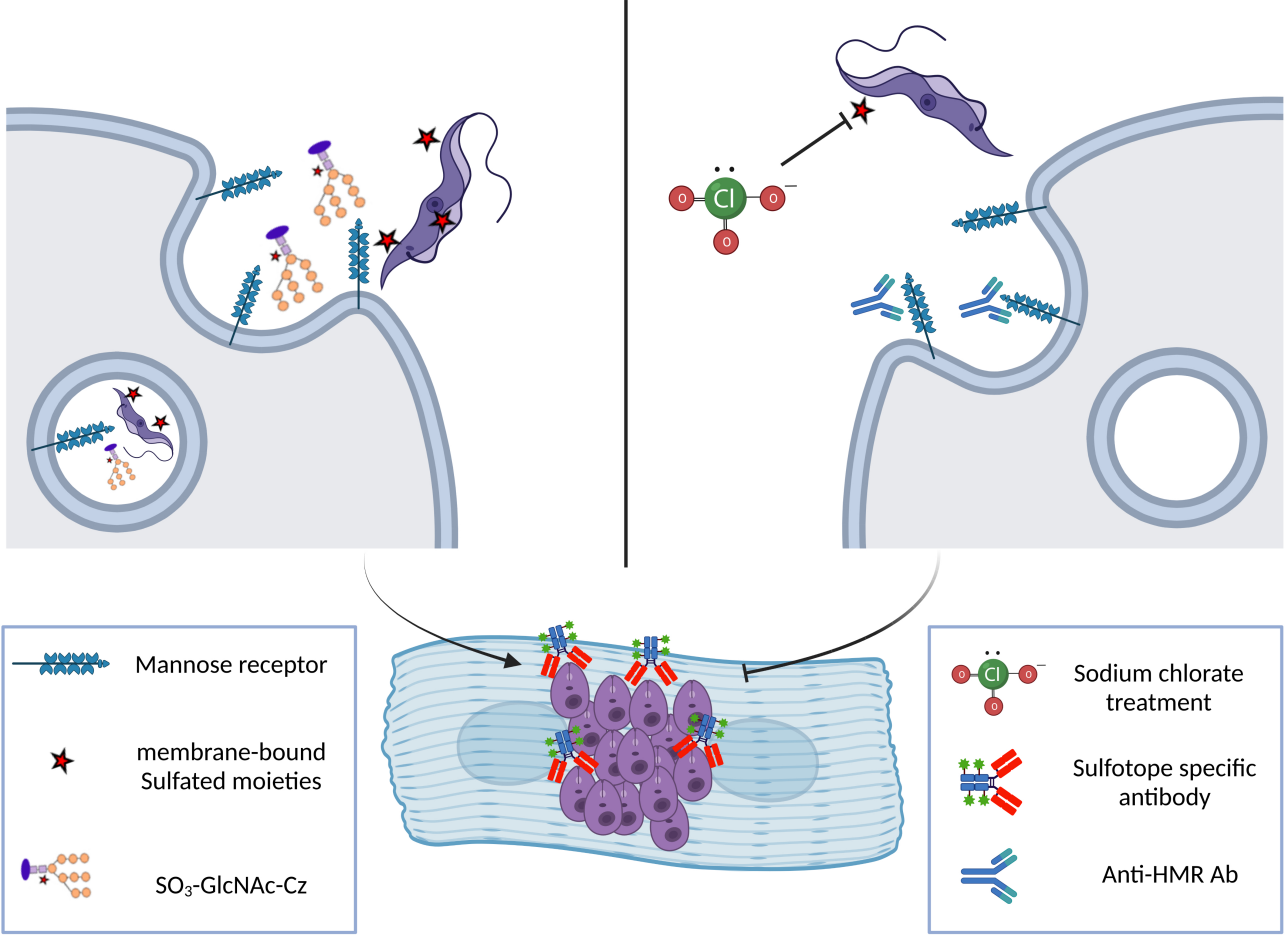
Scheme showing the involvement of Cz sulfotopes in trypomastigote infection of a cardiac cell line. Right: *T. cruzi* trypomastigote enters the target cell *via* HMR-mediated phagocytosis triggered by the interaction of different domains of the receptor with the sulfated glycoconjugates from the parasite. The HMR is a multi-domain protein that presents an N-terminal cysteine-rich region (CR) that recognizes the parasite sulfated sugar residues (Liu et al., 2000). Also, the receptor structure is composed of a fibronectin type II domain (FnII) with unclear function and by a series of eight non-sulfated carbohydrate recognition domains (C-type lectin like CRDs) that recognize terminal mannose, fucose, or NAcGlc residues present in glycosylated molecules on the envelope of pathogen parasites or released glycoproteins. This interaction mediates endocytosis of the trypomastigote form of the parasite during the infection process. Once inside the host cell, trypomastigotes transform into intracellular amastigote, the replicative form. Sulfotopes in trypomastigote and amastigote forms were evidenced using Anti-SO_3_-enriched IgGs. Left: The participation of the mannose receptor and the participation of sulfates from the Cz-C-T domain in the infection process were demonstrated by our group (Ferrero et al., 2014) as treatment with anti-HMR antibodies or anti-sulfotope specific antibodies impairs the infection of cardiac HL-1 cells by trypomastigotes. In addition, the percentage of infected cardiac cells decreased by 40% when using chlorate-treated trypomastigotes. Altogether, these results suggest that sulfation in the parasite contributes to the infection of cardiac cells, and particularly, Cz sulfates and HMR are involved in the infection process by *T. cruzi.*.

Sodium chlorate has been used as an inhibitor to abolish protein sulfation on tyrosine as well as on carbohydrate residues in intact cells. We have examined the *in vivo* effects of sodium chlorate, at different concentrations, on the Cz sulfation mechanism in *T. cruzi* epimastigote forms. Thus, we treated epimastigotes with growing concentrations of sodium chlorate (10–80 mM) for 24, 48, and 96 h. Results showed that, as chlorate concentration increases, parasite Cz recognition by sulfo-specific antibodies decreases, but desulfated Cz recognition, used as a control, was not altered (Ferrero et al., 2014). There are no putative genes for canonical SULTs identified up to now in the *T. cruzi* genome, suggesting that substantial modifications in sequences between mammalian and *T. cruzi* SULTs exist. Therefore, it is necessary to determine if these sequence differences allow these enzymes to be considered valid targets for chemotherapeutic drug design. On the other hand, to begin studies on the sulfation process, epimastigotes were treated with chlorate and analyzed showing Cz undersulfation and a decrease of sulfatide levels, indicating that the *T. cruzi* sulfation mechanism takes place *via* PAPS. In addition, some years ago, we evidenced that trypomastigotes contain surface-sulfated epitopes. Also, trypomastigotes treated with sodium chlorate presented a lower ability to infect cardiac cells at growing chlorate concentrations, suggesting the involvement of Cz sulfotopes in the *T. cruzi* infection process (**Figure 6**, Ferrero et al., 2014).

The exposition of sulfotopes in the trypomastigote surface and their input in the invasion process of the parasite suggest a possible mechanism used by parasites to infect cells (**Figure 6**). In addition to the heightened presence of sulfotopes observed on the surface of the trypomastigote forms, a high recognition was evidenced in a band showing a molecular weight similar to that of the SCP. These results also support the potential involvement of trypomastigote sulfotopes in the parasite–host relationship and/or infection (Ferrero et al., 2014). It is worth mentioning that the sulfation route, parasite SULTs, or both might be relevant targets for chemotherapy of ChD and are under study by our research group.

### 1.7 Sulfotopes from cruzipain interact with the immunomodulatory molecule Siglec-E

**Figure 7:**
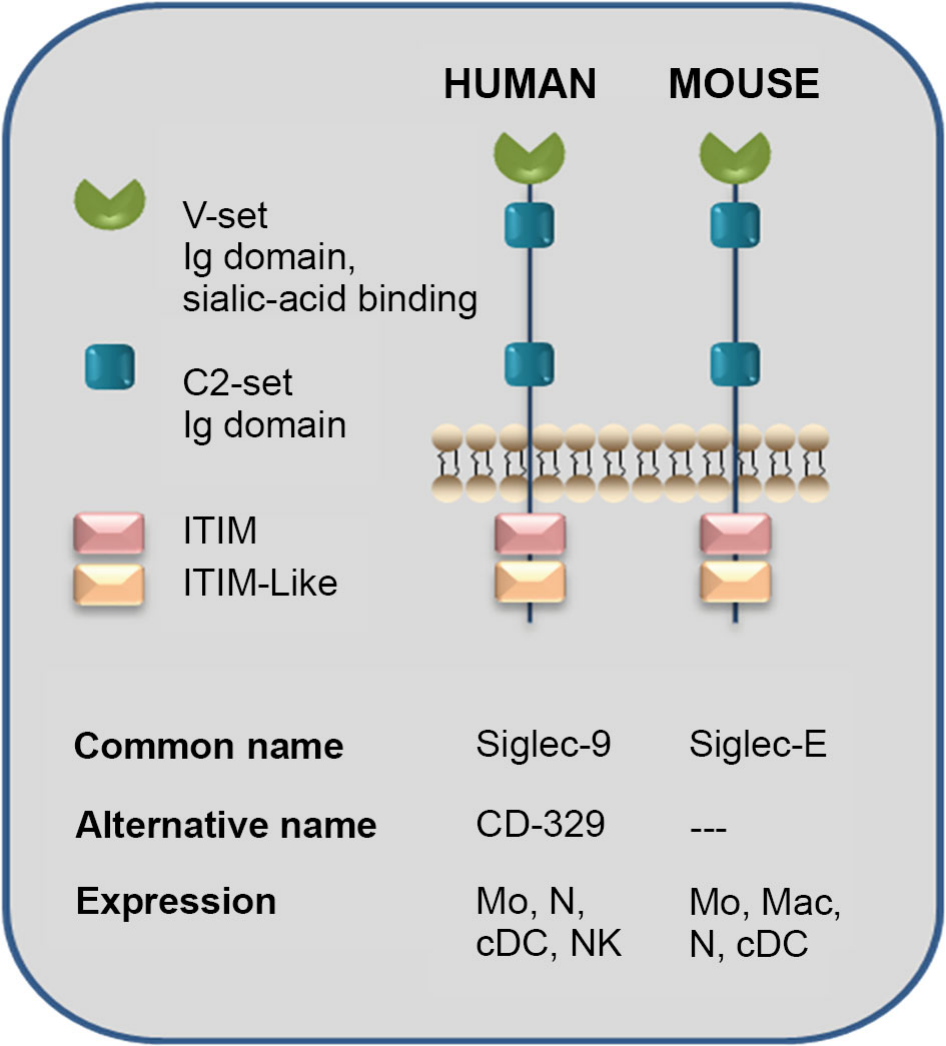
Sialic-acid-binding immunoglobulin-like lectins (Siglecs): Human Siglec-9 and Mouse Siglec-E structure. The human Siglec-9 structure is composed of a single V-set Ig domain, which confers glycan specificity, and two C2-set Ig domains at the extracellular region connected through a transmembrane linker to a cytoplasmic tail containing an immunoreceptor tyrosine-based inhibition motif (ITIM) domain and an ITIM-like one. The expression cells are neutrophils, monocytes, dendritic cells, and natural killer cells and present a particularly strong affinity for sialylated glycans containing a GlcNAc_6_SO_3_ although non-sulfated sialylglycans and even hyaluronic acid are recognized by this lectin with weaker affinity. An orthologous protein of human Siglec-9 is murine Siglec-E, which may have comparable roles in the immune system and share similar cell-type expression. Even though Siglec-E presents binding selectively to sialic acid itself, we have demonstrated that sulfation increases Siglec-E recognition of N-glycans present in Cz (Ferrero et al., 2016). Mo, monocytes; Mac, macrophages; N, neutrophil; cDC, conventional dendritic cell; NK, natural killer (adapted from Crocker et al., 2007).

Siglecs (sialic-acid-binding immunoglobulin-like lectins) are type 1 membrane proteins found in humans and rodents, which recognize sialic acid-containing ligands, and exert varied immuno-modulatory effects. The structure of human Siglec-9 is composed of a single V-set Ig domain, which confers glycan specificity, and two C2-set Ig domains, at the extracellular region, coupled through a trans-membrane linker to a cytoplasmic extension that holds an immunoreceptor tyrosine-based inhibition motif (ITIM) domain and an ITIM-like domain (Crocker et al., 2007). Its expression is mainly in neutrophils, monocytes, dendritic cells (DCs), and natural killer cells, among others, and it exhibits a strong affinity for sialylated glycans containing a GlcNAc_6_SO_3_. Non-sulfated sialoglycans and even hyaluronic acid are also recognized by this lectin, but with weaker affinity (Yu et al., 2017). Murine Siglec-E, an orthologous protein of human Siglec-9, shares a similar expression pattern and appears to make certain comparable properties in the immune system components with the mentioned one (McMillan et al., 2013). The structure and characteristics of Siglec present in humans and mice are shown in **Figure 7**.

On the other hand, *T. cruzi* transialidases (TS) cleave sialic acid from host cell glycoproteins and transfer them to mucin-like structures on the parasite, which may confer protection against the immune system. Indeed, increased TS activity is associated with strain pathogenicity. Previous studies using Siglec-E-expressing CHO cells showed that *T. cruzi* trypomastigotes from the pathogenic Tulahuen strain recruit Siglec-E to the binding site on the plasmatic membrane; instead, when the non-pathogenic Tehuantepec strain was used, there was no Siglec-E recruitment. These results were associated with the increased TS activity in the Tulahuen strain, which also presented more Siglec-E binding than the Tehuantepec strain. Moreover, antibody cross-linking of Siglec-E on DCs decreases IL-12 production in response to lipopolysaccharide (LPS). Interestingly, the Tulahuen strain was capable of reducing IL-12 and increasing IL-10 production in LPS-stimulated DCs in a sialic-acid-dependent way (Jacobs et al., 2010).

Further studies on Siglec-E interaction with *T. cruzi* performed by our group showed that membrane and secretion proteins account for most Siglec-E ligands in the parasite. Among them, Siglec-E binds to Cz molecules secreted by metacyclic trypomastigotes, as well as lysosomal Cz from *T. cruzi* epimastigotes. Cz sulfation enhances Siglec-E recognition since desulfation treatment over Cz reduced its interaction with Siglec-E as observed by ELISA. Sulfation contribution to Siglec-E recognition was also confirmed in trypomastigotes, where chlorate treatment decreased Siglec-E recognition. Together, these findings evidence that the sulfotope-containing sialylated glycoproteins from *T. cruzi* could contribute to host immuno-modulation through their interaction with Siglec-E, favoring parasitemia and the persistence of the parasite (**Figure 7**). Whether this mechanism is also present in natural human infection remains to be studied (Ferrero et al., 2016).

### 1.8 Association between sulfotopes and immunopathogenesis of experimental Chagas disease

**Figure 8:**
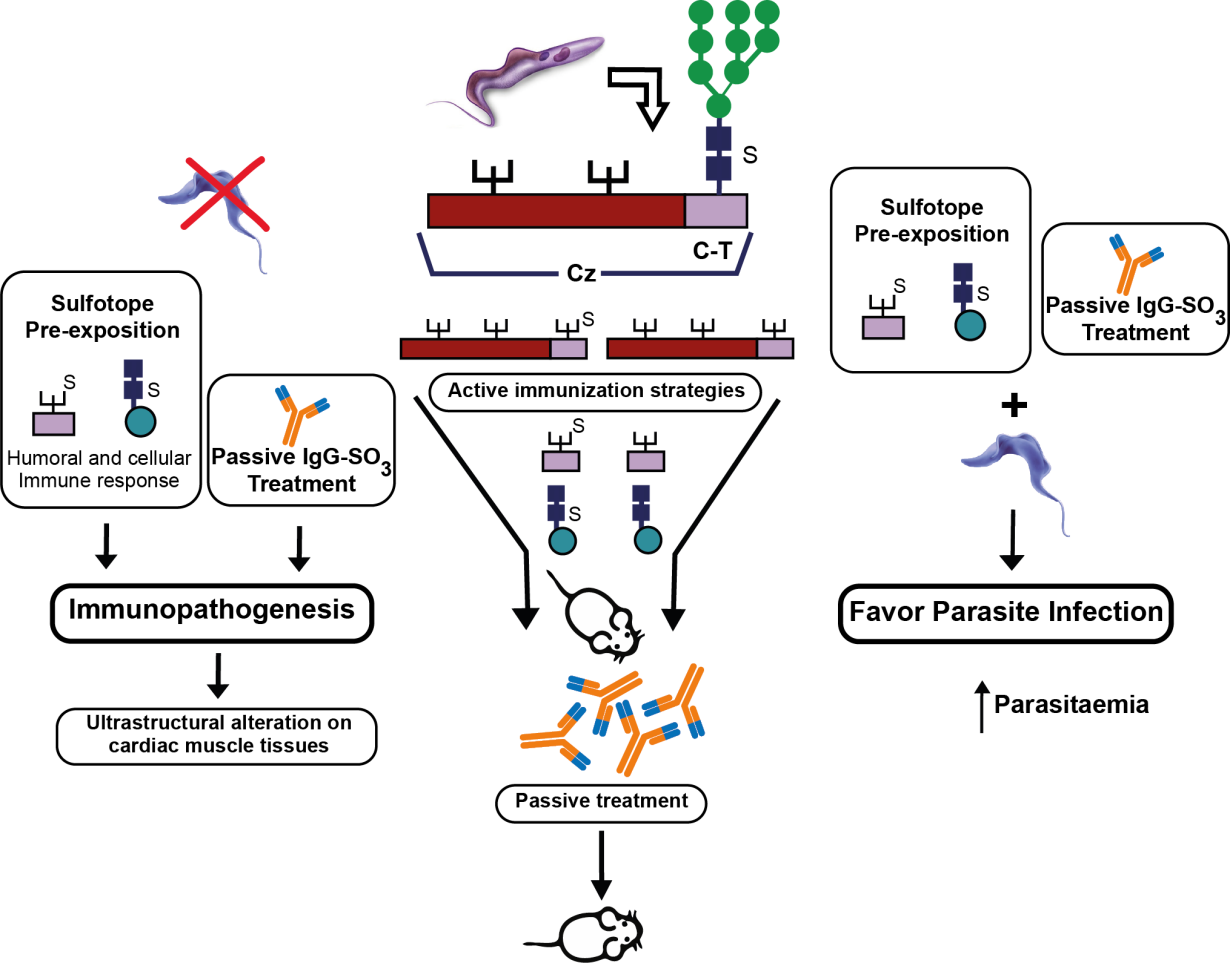
Participation of (i) sulfotopes and (ii) specific antibodies for sulfotopes: both in the immunopathogenic process and in the infection by *T. cruzi*. The scheme resumes the input of sulfotopes and their specific antibodies in the immunopathology by directing the tendency of the immunological host response when confronted with the parasite, by the cross-reactivity between Cz and other muscle proteins, particularly myosin, and in the mechanisms of invasion and virulence of the parasite throughout the serious acute phase of infection. In the middle of the figure, the scheme shows the Cz, the C-T, and the three glycosylation sites, one in the C-T representing the sulfate with an S. The three strategies of active immunization with Cz, C-T, and the sulfotope are shown, as well as the passive immunization with antibodies specific for sulfotopes. On the left part of the figure, it can be observed that in the absence of infection, the sulfotopes’ pre-exposition that determines an immune response and the passive treatment with antibodies specific for sulfotopes determine immunopathogenesis with the consequent characteristics on ultrastructural alterations on cardiac muscle tissues. On the right part of the figure, it can be observed that the sulfotopes’ pre-exposition and the passive treatment with antibodies specific for sulfotopes, followed by challenge with trypomastigotes, favor parasite infection, thus increasing parasitemia (Soprano et al., 2022).

Although we found some biological roles of *T. cruzi* sulfated structures in the infection and the immunomodulation processes (Ferrero et al., 2014; Ferrero et al., 2016), the participation of sulfotopes (GlcNAc_6_6SO_3_) in the immunopathogenesis of experimental ChD still needs to be completely elucidated. In this sense, preliminary results of our laboratory have demonstrated the involvement of sulfotopes from *T. cruzi* glycoproteins and their specific antibodies in the immunopathogenicity observed in host–tissue interaction during experimental ChD giving preference to *T. cruzi* infection (Soprano et al., 2018b; Duschak et al., 2020). Recently, to further clarify the effects of GlcNAc_6_SO_3_ on the selected murine model, BALB/c, three independent approaches were carried out by our research group: (i) immunization with purified C-T obtained from the Cz molecule; (ii) use of the synthetic GlcNAc_6_SO_3_ coupled to BSA as immunogen; and (iii) treatment with purified IgGs-GlcNAc_6_SO_3_. The strat of immunization were tailed by an under-lethal defy with *T. cruzi* trypomastigotes (**Figure 8**). The choice of sub-lethal dose was due to avoid mice mortality in order to analyze the immunopathogenesis is process that occurs in post-acute phase on target tissues pre-exposed to sulfotopes. The murine model was selected because of its susceptibility and auto-reactivity, and it has been previously demonstrated as an outstanding archetype since (a) its immune response profile to *T. cruzi* infection has long been established to be well-known; (b) the murine strain selected has the ability to imitate both phases, acute and chronic phase models (Cardoni et al., 1999); (c) along the acute phase, it survives increased parasitemia (Cardoni et al., 1999; Wrightsman et al., 1982); (d) this strain constitutes a chronic model, which allows one to cautiously examine tissue pathogenesis in the chronic phase (Cardoni et al., 1999; Roggero et al., 2002; Soprano et al., 2022); and (e) simply, the selected female mice are more resistant to the progress of infection by *T. cruzi*. Despite the fact that, in *T. cruzi* infection, the strain does not present as marked polarization as it was observed in Leishmaniasis, BALB/c mice have a biased response towards Th2 (Huang et al., 1997; Kuroda et al., 2002). Nevertheless, it has been reported that during the acute phase of *T. cruzi* infection, at the splenic level, the immune response associated with the parasite’s initial control is Th1 (Cardoni et al., 1999; Rostamian et al., 2017). Accordingly, cytokines’ proposed splenic profile obtained in mice immunized with C-T could be attributed to the involvement of parasite sulfotopes. Anti-sulfated moieties, which showed cross-reactivity with a variety of self-antigens present in the heart (Acosta et al., 2008) and possibly in additional muscle tissues, were exhibited in sulfotope-containing antigen-immunized mice and exposed to experimental infection by *T. cruzi*. In line with this, deposits of IgGs located on heart myofibrils in mice immunized with Cz (Giordanengo et al., 2000) suggest the involvement of IgGs-GlcNAc_6_SO_3_, generating ultra-structural heart abnormalities. We have shown that a model using BALB/c as mice/Tul2-*T. cruzi* has been established and it is highly suitable (**Figure 8**).

### 1.9 Glycosphingolipids

Glycosphingolipids are ubiquitous compounds in eukaryotic cells. Among them, gangliosides and sulfoglycosphingolipids (SGSLs, also known as sulfatides) representing acidic glycosphingolipids (AGSLs) develop relevant roles such as participation in adhesion procedures, in proliferation, and also in the differentiation of several cellular systems. Regarding SGSLs, they are biosynthesized by the action of SULTs that link the sulfate groups as monoesters to the sugar moiety. Two different enzymes have been described: the one producing HSO_3_-3GalCer and HSO_3_-3LacCer, and GlcA-3-O SULT, responsible for the Human Natural Killer-1 (HNK-1) sulfated (HSO_3_-3GlcAβ1-3Galβ1-4GlcNAc-R) epitope synthesis (Senn et al., 1990; Bakker et al., 1997; Honke et al., 1997; Hirahara et al., 2000). Although these lipids were first identified in brain tissue, they have been detected in the kidney, gastrointestinal tract, islet of Langerhans, trachea, and cancer cell lines. As cellular sulfatide levels change, they influence cardiovascular and cancer disease progression and have been proposed as potential biomarkers (Xiao et al., 2013). Interestingly, SGSLs are also biosynthesized by parasitic protozoa. They have been evidenced in the intraerythrocyte stages of *Plasmodium falciparum* (Landoni et al., 2007) and in the different *T. cruzi* developmental forms (Barreto-Bergter et al., 1985; de Lederkremer et al., 1985; Uhrig et al., 1992).

#### 1.9.1 Sulfoglycosphingolipids in *Trypanosoma cruzi*

Our group performed the first study on the extraction, purification, and structural characterization by UV-MALDI-TOF-MS analysis of SGSLs existing in epimastigote developmental stages of *T. cruzi* parasites. Sulfoglucuronyl-containing dihexosylceramides mostly composed of sphingosine as the extended chain base acylated with stearic acid were determined (Acosta et al., 2012). Regarding the role of these types of acidic lipids, sulfated glycolipids acting as common antigens between *T. cruzi* and mammalian tissues were previously described (Petry et al., 1988). Later, neutral glycosphingolipids (GSLs) were also involved as antigens responsible for the cross-reactivity in the autoimmunity existing in ChD (Vermelho et al., 1997). However, scarce information was accessible on SGSL structural constructions and their role in protozoan flagellate parasites (Leipelt et al., 2001).

**Figure 9:**
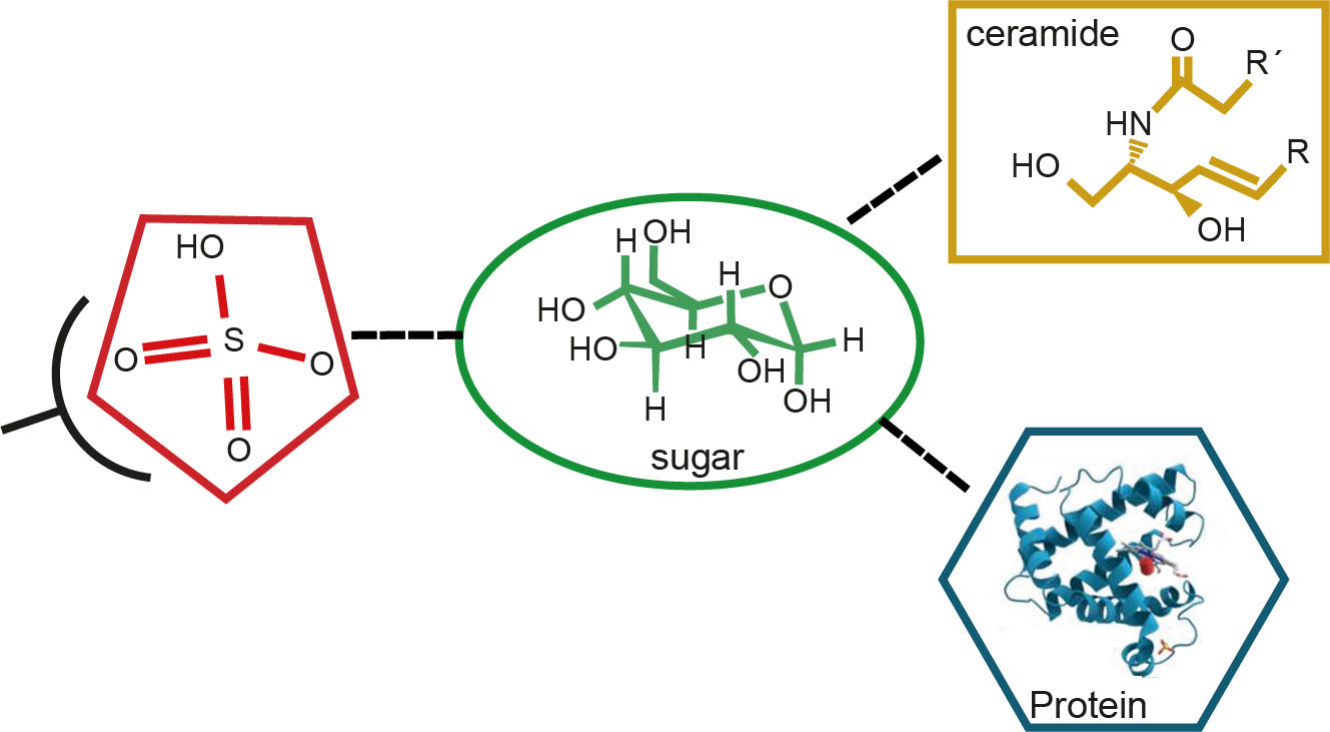
Scheme of the antigen–antibody reaction of sulfate groups from sulfotopes (sulfate plus sugar) linked to glycolipids or glycoproteins. The scheme describes the structure of a sulfoglucuronyl-containing sphingolipid of *T. cruzi* previously determined by UV-MALDI-TOF-MS analysis, mainly composed of d20:1-C18:0 ceramide. We have evidenced that sulfate is an epitope shared by both the C-T domain of Cz and the sulfatides from *T. cruzi.* Also, we have shown that there are other sugar epitopes in the glycolipidic structures participating in the studied cross-reactivity. Moreover, we have demonstrated that raised levels of human anti-IGg_2_ antibodies specific for sulfotopes present in sulfatides resulted in correlation with the forms showing low severe disease in chronic ChD patients.

In line with this, we performed immunologic inhibition assays with specific anti-Cz and anti-C-T antibodies, after developing a procedure of adsorption with growing quantities of sulfatides obtained from epimastigotes, before and after desulfation, demonstrating that the sulfotope is shared between Cz and sulfatides of *T. cru* ***zi***. It was also determined that the cross-reactivity was located in the C-T domain of Cz. Furthermore, a comparison of sera from *T. cruzi* chronic infected individuals with mild disease, belonging to G0/G1 groups, with those of people suffering from severe disease forms from groups G2/G3 showed higher levels of IgG_2_ antibodies for sulfatides in the former group. Therefore, we have provided evidence that *T. cruzi* sulfate groups individually represent antigenic epitopes of the kind of glycoconjugate, which is a sulfated glycoprotein or a sulfatide, suggesting that antibodies specific for sulfated groups are associated with protective immunity and with mild chronic ChD, which might be a predictor of stability in the initial periods of the disease (**Figure 9**) (Acosta et al., 2012). Finally, we have provided evidence that sulfate moieties from *T. cruzi* associated with carbohydrates (sulfotopes) proved to be antigenic and that the sulfotope-specific antibodies for glycoconjugates, IgG_2_, might be well thought out as biomarkers of the progression of chronic ChD.

## 2 Conclusion

Our research findings have demonstrated that (i) the preferred epitope for the immune recognition is constituted by a glucosamine bearing a sulfate group in position O-6 and an N-acetyl group; (ii) the synthetic GlcNAc_6_SO_3_ mimics the N-glycan-linked sulfotopes that are displayed in the natural C-T domain of the Cz, which supports the influence of this sulfated sugar in the human humoral response; (iii) *T. cruzi* glycoconjugates sulfotopes show antigenic properties; (iv) sulfotopes are connected with immune cross-reactivity between the two sulfated glycoproteins described in *T. cruzi*, SCP, and Cz; (v) there is cross-reactivity between sulfatides and Cz; (vi) the exposition of sulfotopes in trypomastigote surface supports their input in parasite–host relationship and/or in the invasion process of the parasite, suggesting a possible mechanism used by parasites to infect cells; (vii) the binding of sulfosialylated glycoproteins to sialic-acid Ig-like-specific lectins (Siglec) by host–parasite interaction indicates the participation of Cz sulfotopes and those from other sulfated glycoproteins in the immunomodulation process; (viii) sulfotopes are involved in the generation of cardiac injury in BALB/c mice, in the absence of infection; (ix) *T. cruzi* sulfotopes and antibodies specific for sulfotopes have shown a central effect in the host–tissue immunopathogenicity along experimental ChD as well as in the infection by *T. cruzi*; and (x) sulfotope-specific human IgG_2_ antibody levels are inversely correlated with ChD severity.

Remarkably, sera from chronically *T. cruzi*-infected subjects with mild disease exhibited higher levels of IgG_2_ antibodies specific for sulfotopes from sulfated glycoconjugates (glycoproteins and sulfatides) compared to those with more severe forms of the disease, demonstrating that *T. cruzi* sulfotopes are antigenic irrespective of the kind of glycoconjugate. Ongoing trials point to sulfotope-specific antibodies as potential predictors of stability from mild early stages of chronic coronary disease that could be considered biomarkers of human heart disease progression. In addition, taking into account the presence of sulfated glycoproteins in the parasite, the absence of SULTs described so far in *T. cruzi*, and the lack of sequences homologous to SULT motifs in the predicted protein database from *T. cruzi* genome, the identification of novel parasite pathways and/or the SULTs involved in the biosynthesis of sulfated structures presenting differences with those described in mammals might be used as alternative therapeutic targets. Thus, a detailed study on the proposed roles for sulfotopes from Cz and/or other potential sulfated glycoproteins and SULT(s) will help to understand the biosynthesis sulfation route still unknown in Trypanosomatids and the mechanisms of immunopathogenicity and infection of these organisms and eventually to define a new chemotherapeutic target for the treatment of ChD as well as potential biomarkers of cardiac disease progression.

## Author contributions

The section related to glycobiology and post-translational modifications on glycoproteins (Cz and SCP) and glycosphingolipids was written by AC and VD. The section corresponding to sulfotopes, Siglecs, and immunomodulation was originally written by TJ and VD. The section related to immunopathology was accomplished by LS and VD. Figures were designed by VD, LS, MF, and AC and executed by Claudia Nose. The general idea of the review was conceived by VD. All authors contributed to the article and approved the submitted version.
